# Radiosensitization of orthotopic GIC-driven glioblastoma by doxycycline causes skin damage

**DOI:** 10.1186/s13014-019-1266-4

**Published:** 2019-04-08

**Authors:** Guido Frosina, Daniela Marubbi, Diana Marcello, Antonio Daga

**Affiliations:** 1 Mutagenesis & Cancer Prevention, IRCCS Ospedale Policlinico San Martino, Largo Rosanna Benzi 10, 16132 Genoa, Italy; 2 Cell Oncology, IRCCS Ospedale Policlinico San Martino, 16132 Genoa, Italy; 3 Department of Experimental Medicine (DIMES), University of Genova, 16132 Genoa, Italy

**Keywords:** glioma animal models, radiosensitization, doxycycline

## Abstract

Doxycycline (DXC) is a tetracycline antibiotic which has been proposed as a breast cancer radiosensitizer by specifically reducing the expression of the DNA repair enzyme DNA PK in breast cancer initiating cells. Since DXC presents favorable pharmacokinetics properties including the capacity to cross the blood-brain barrier, it has been hypothesized that it could radiosensitize brain tumors as well. We have investigated the radiosensitizing capacity of DXC towards human glioma initiating cells (GIC)-driven orthotopic glioblastomas (GB) in NOD/SCID mice that faithfully mimic the growth properties of the clinical tumors of origin. DXC at 10 mg/Kg body weight was subcutaneously delivered daily, 5 days/week for 4 weeks. At the same time, radiotherapeutic fractions of 0.25 Gy to the head were delivered every 3–4 days (twice/week) for 15 weeks. No survival advantage was observed in DXC-treated mice as compared to vehicle-treated mice by this radiosensitizing protocol. On the contrary, skin damage with hair loss and deep ulcers were observed after 4 weeks in DXC-treated mice leading to discontinuation of drug treatment. These results do not support the use of DXC as a radiosensitizer for brain tumors and indicate skin damage as an important side effect of DXC.

## Background

Glioblastoma (GB-WHO grade IV) is the most common malignant brain tumor in adults; it is almost invariably lethal in 10–12 months. GB appears as infiltrating lesions on MRI or CT often characterized by central necrosis, perilesional edema and abnormal vasculature [1]. Radiotherapy for primary GB involves a total ionizing radiation (IR) dose to the tumor of 54–60 Gy given in 1.8–2 Gy fractions 5 days/week. The tumor usually relapses in a few months after which re-irradiation is in most cases ineffective. Resistance to IR may be linked to specific tumor cell populations often (but not invariably) displaying stem properties (glioma initiating cells – GIC) [2, 3].

Since its FDA-approval in 1967, doxycycline (DXC) has been used as a broad-spectrum antibiotic targeting bacterial ribosomes. In mammalian cells, DXC may function as an inhibitor of mitochondrial biogenesis by binding to the small subunit of the mitochondrial ribosome which shows a number of conserved properties and protein homologies with ancestor bacterial ribosomes [4]. Clinical trials with DXC serendipitously showed positive therapeutic effects in lymphoma patients [5, 6], generating the hypothesis that DXC treatment may specifically reduce the oxidative mitochondrial capacity and the glycolytic activity of cancer cells [7]. DXC has been further reported to reduce in breast tumor initiating cells the expression of the DNA-PK protein, which is involved in DNA repair of IR-induced damage. Consistently, the cells were radio-sensitized up to 4.5-fold [7, 8]. DXC has shown favorable pharmacokinetics properties, with nearly 100% oral absorption, an 18–22 h serum half-life and the capacity to cross the blood-brain barrier, thus suggesting its use as a radio-sensitizer of brain tumors. We have investigated here the radio-sensitizing capacity of DXC using a GIC-driven orthotopic mouse model of GB.

## Methods and materials

### Animals, DXC

Mice were provided by the Breeding Unit of the Animal Facility at IRCCS Ospedale Policlinico S.Martino - Genova, Italy. Immunodeficient non-obese diabetic/severe combined immunodeficient (NOD-SCID) mice were housed under maximum barrier conditions, one animal per cage to facilitate health status inspection. Husbandry conditions included a 12 hours fluorescent light/dark cycle and ad libitum access to standard laboratory chow and water. The experimental unit was the single animal that was uniquely identified by a number marked on the tail and coded by ear punches. This mouse ID number was in the format XX.XX where the first two digits indicate the experiment and the second two digits indicate the animal. The ARRIVE (Animal Research: Reporting of In Vivo Experiments) guidelines were followed throughout this report [9].

One 100 mg tablet of DXC [Bassado (Pfizer Italia, Latina, IT)] was dissolved in 100 ml of 0.9%NaCl under stirring (f.c. 1 μg/μl). The solution was then filtered, divided into aliquots and frozen at –20°C. Each aliquot was thawed only once.

The pure formulation of the drug was purchased from Sigma (doxycycline hyclate – cat D9891) and dissolved as above.

### The orthotopic COMI GB

**Fig. 1:**
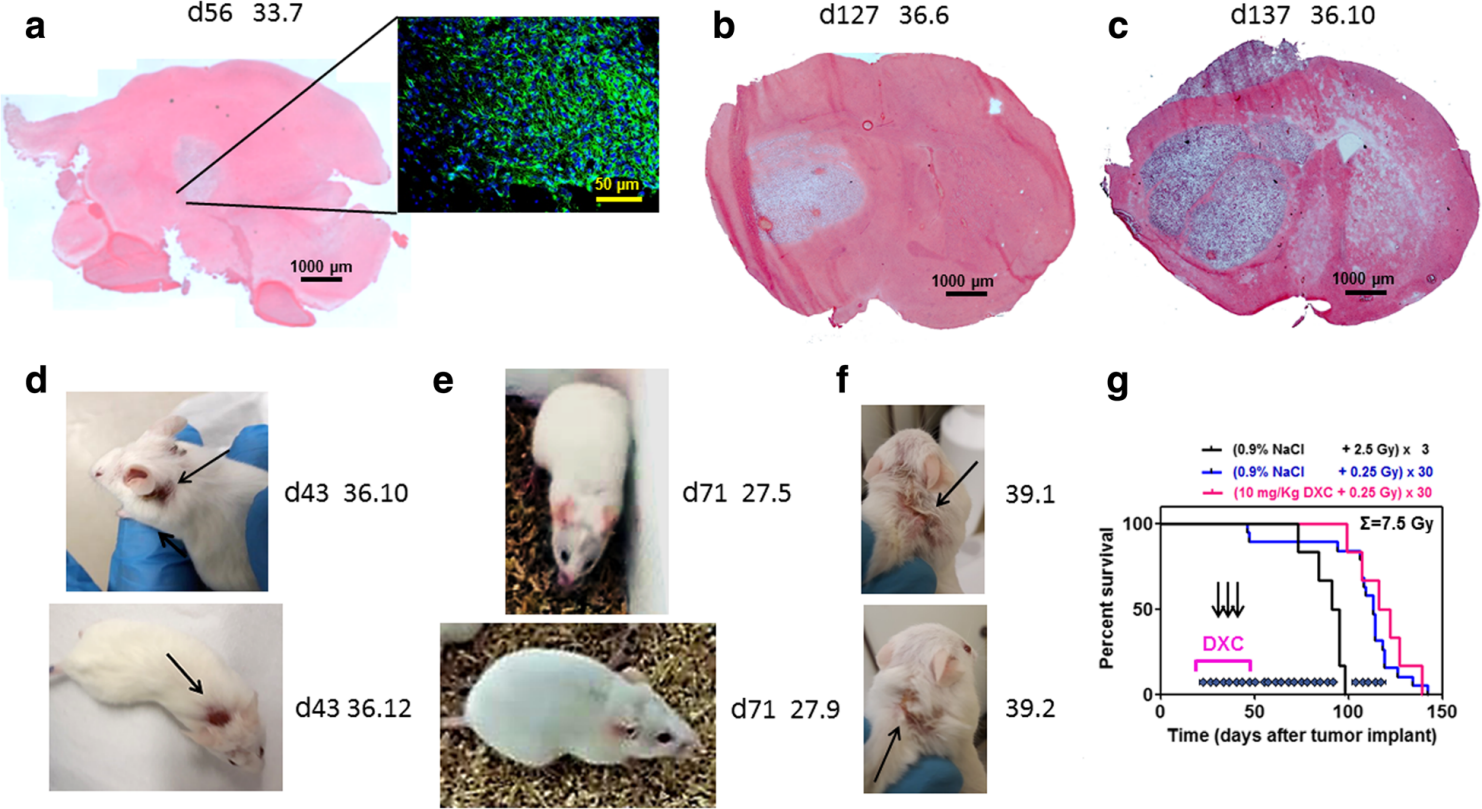
Skin damage to NOD-SCID mice bearing orthotopic glioblastoma radiosensitized by chronic DXC administration. **a**-**c** H/E staining of brain sections taken at d56, d127 and d137 of tumor development showing progression of orthotopic COMI glioblastoma induced to NOD SCID mice. Immunostaining at d56 (Fig. **a**-inset) reveals that most of the orthotopic tumor expresses the stem cell marker nestin, indicating its stem cell-driven character. Mouse ID numbers 33.7, 36.6 and 36.10 are indicated for the sake of reference. **d**, **e** Skin damage to the shoulders region of DXC-treated mice at d43 of tumor development. DXC was subcutaneously administered daily at 10 mg/kg on weekdays starting d21 of tumor development (**d**). Control mice were administered with vehicle only (0.9% NaCl) (**e**). 0.25 Gy of ionizing radiation were delivered to the head of all mice twice a week (Tuesday and Friday) starting at d22 and ending at d130 of tumor development. DXC administration was discontinued at d49 of tumor development due to the deep skin ulcers appearing in DXC-treated mice. **f** Skin damage to the shoulders region of mice treated with a pure formulation of DXC. 60 mg/kg DXC was subcutaneously administered once on the first day of treatment followed by 10 mg/kg DXC twice daily for a total of 7 days. **g** Kaplan-Meier survival curves of irradiated animals. Median survival of DXC-treated animals (magenta) was not significantly different from that of vehicle-treated controls (blue) (119 vs. 113 days; ratio: 0.949; P: 0.445). The latter survived significantly longer in comparison to mice treated with standard 3 × 2.5 Gy fractions (black) (113 vs. 93 days; ratio: 1.215; P: 0.0009)

The primary GIC line COMI derived from an adult GB has been previously described [10–13] (Fig. 1a-c). Its authentication was performed by determining the proliferation rate, the expression of DDR, stem, *PI3K/Akt* pathway genes as well as the *IDH1, TP53, H3F3A, PDGFRA, CDKN2A* and *EGFR* status as previously described [10–13]. COMI GICs have wild type *TP53* sequence but they express the TP53 RNA at low levels [11]. COMI GICs initiate orthotopic glioma development with > 95% efficiency when injected i.c. into immunodeficient NOD-SCID mice [10–13] (Fig. 1a-c). To this aim, 4-5 weeks old NOD/SCID mice were anesthetized with isoflurane. Thereafter, the animals were positioned into a stereotaxic frame (David Kopf instruments) and a hole was made, using a 21-gauge needle, 2.5 mm lateral and 1 mm anterior from the intersection of the coronal and sagittal sutures (Bregma). 1.3 x 10^5^ GIC were injected at a depth of 3 mm in correspondence of the left corpus striatum and the skin closed using metal staples (Martin GMBH, Tuttingen, Germany). In order to avoid significant subpopulation selection during prolonged cell culture, GIC samples frozen after no more than 30 days of culture were used for orthotopic tumor development.

### Experimental design

Twelve mice were randomly assigned to the experimental (six animals) and control (six animals) groups: mice in the experimental group were subcutaneously (s.c.) infused in the dorsal region over the shoulders on weekdays (5 days per week) for 4 weeks (from d21 to d46 of tumor development) with 10 mg/Kg DXC in 0.9% NaCl. Animals in the control groups were s.c. infused with an equal volume of vehicle.

RT of orthotopic GB was performed by an RS 2000 Biological Irradiator (Rad Source Technologies, Alpharetta, GA, USA [13]) whose “in vivo” delivered dose was verified by a RadCal Accu-Gold system (Monrovia, CA, USA) equipped with a 10X6-0.6 High Dose Rate Chamber [13]. The dose was confirmed by two radiochromic films (Gafchromic® EBT3, Ashland Inc., Covington, KY, USA) placed over and under the mouse body [13]. An iperfractionated radiation schedule was adopted with a total final dose of 7.5 Gy delivered in thirty 0.25 Gy fractions from d22 to d130 of tumor development, one fraction every 3-4 days (Fig. 1g). In the days of DXC administration, IR was delivered 5 h after the drug. In order to reduce toxicity of anesthesia during repeated drug administrations and RT, an isoflurane inhalation anesthesia apparatus was used.

Animals were monitored daily in blind by experienced researchers assigning to each animal a health score (H1 moribund status – H10 full health) based on neurological symptoms including posture changes, arched back, tail weakness, diminished activity and skin turgor. Euthanasia was performed by CO_2_ asphyxiation at the moribund status H1 that was the primary experimental outcome in this study.

For histological analysis, animals were euthanized by CO_2_ asphyxiation and brains were cryopreserved. Coronal sections obtained at a cryostat microtome were fixed and stained with hematoxylin/eosin (H/E) or nestin.

## Results

### The GIC-driven COMI tumor

We wished to investigate whether DXC may radiosensitize GIC-driven orthotopic tumors that faithfully mimic the growth patterns of clinical tumors. The GIC driven COMI tumor and its histological and growth features closely resembling the original patient’s tumor have been described [13]. Briefly, under matrigel-coating and serum-free conditions, COMI GICs slowly grow and layer into a monolayer, maintaining intact self-renewal capacity. The stem cell marker nestin is expressed at high levels in COMI GIC (Fig. 1a). In the absence of matrigel, COMI GICs predominantly grow forming suspended neurospheres. Removal of growth factors and addition of 10% FCS to the proliferation medium, after approximately 2 weeks induce GIC differentiation with acquisition of astrocytic morphology, altered refractory index, and increased expression of a number of differentiation markers including GFAP and beta III tubulin [10, 14].

1.3 x 10^5^ COMI serum-free grown GIC were stereotactically injected into the left corpus striatum of NOD/SCID mice. Staining with hematoxylin/eosin of brain tissue sections revealed 56 (Fig. 1a), 127 (Fig. 1b) and 137 (Fig. 1c) days later a voluminous progressing expansive lesion with an irregular infiltrating wall that exerted considerable mass effect on the adjacent structures (Fig. 1b, c).

### Skin damage by DXC

In order to investigate the radiosensitizing capacity of DXC, twelve mice bearing the orthotopic COMI tumor were treated with total 7.5 Gy to the head delivered in 30 x 0.25 Gy fractions from d22 to d130 of tumor development (Fig. 1g). Each fraction was delivered every 3-4 days (twice/week). To obtain reproducible and complete engraftment of GIC-driven orthotopic gliomas, GICs had to be injected in radiosensitive NOD SCID mice that present a suitable level of immunodeficiency as compared to more radioresistant, but less immunodeficient strains such as nude mice [15]. Previous studies, while showing the beneficial effect on these animal models of collimated radiation to the tumor, also showed increased radiotoxicity in those animals for cumulative doses higher than 7.5 Gy and complete lethality for doses higher than 10 Gy [13]. On the basis of those observations, a cumulative radiation dose of 7.5 Gy was chosen in the present experiments. This dose was delivered in 30 x 0.25 Gy fractions to contain the cumulative toxic effects of radiation and DXC and on the basis of previous studies showing that the beneficial effect of radiosensitizing agents on animal survival is increased by adopting a hyperfractionated radiation schedule [13].

Six mice in the experimental group were subcutaneously injected on weekdays in the dorsal region over the shoulders, with 10 mg/kg DXC starting d21 of tumor development. Six control mice were administered with vehicle only (0.9% NaCl). DXC administration had to be discontinued at d49 of tumor development due to deep skin ulcers appearing on the back of DXC-treated mice, more frequently in proximity of the injection sites (Fig.1d,e). Similar skin damage was observed in unirradiated mice treated with a pure formulation of DXC, indicating that the skin damage was specifically caused by the drug itself rather than its combination with IR or other components present in the Bassado tablets (Fig. 1f).

Albeit the presented results arise from a single experiment, they might indicate likely skin damage complications by DXC that were observed in all mice in the DXC-treated group as compared to none in the vehicle-treated group. No significant difference in overall survival was observed between DXC-treated and control mice under those conditions (Fig. 1g). In the absence of drug, preliminary results indicate significant improvement of survival in animals irradiated with an hyperfractionated radiation schedule (0.25 Gy x 30) as compared to animals irradiated with standard fractions (2.5 Gy x 3) (Fig. 1g).

## Discussion

Damage has been occasionally described to different tissues of DXC-treated patients. A challenging case of a 13-year-old adolescent boy who acutely developed ulcerative plaques as well as systemic symptoms after being treated with DXC and isotretinoin for acne conglobata has been reported [16]. After sunlight exposure, clinical skin symptoms in DXC-treated patients may vary from light sunburn-like sensation (burning, erythema) to large-area photodermatitis [17].

Gastric mucosal toxicity has been reported after oral use of DXC. Gastric mucosal erosions covered with adherent exudate have been repeatedly observed by esophagogastroduodenoscopy in patients assuming DXC orally [18, 19]. Microscopic analysis of biopsies from the gastric lesions revealed superficial mucosal necrosis and capillary vascular degeneration, usually resolving upon discontinuation of the drug.

Parenteral administration of DXC may cause varying damages as well. Injectable DXC formulations (trade names: Vibramycin, Pfizer USA; Vibrovenös, Pfizer Switzerland) are intended for intravenous administration and, if infused intramuscularly, may cause muscle necrosis at the site of injection, more severe with the U.S. formulation [20].

Using a faithful mouse model of human GB where orthotopic tumors were induced by primary GICs, we found no beneficial effect of subcutaneously administered DXC in terms of overall survival to animals treated with whole brain radiotherapy. For radiosensitization, the effect of the radiosensitizing drug has to be present during the DNA repair phase [8]. This phase is starting directly after irradiation and lasts approximately 12-24h, depending on cell type [21]. Since the half-life of DXC in the body is 18-22 hours, its administration 5 hours prior to each radiation fraction would have allowed an adequate DXC level during the DNA repair phase occurring in tumor cells. No improvement of animal survival was yet observed.

We have recently shown that adoption of a hyperfractionated irradiation scheme increases the radiosensitizing capacity of ATM inhibitors towards the COMI GIC-driven orthotopic tumor model [13]. The radiosensitizing effect linked to the inefficient activation of the G2/M checkpoint by low-dose irradiation schemes [22, 23] partially summed to the radiosensitizing effect of inhibition of residual ATM activation, with an overall improved beneficial effect on animal survival [13].

ATM and DNA-PKcs are core factors of the DNA damage response (DDR), a common resistance mechanism in GIC [24, 25]. They share common domain organization, structural similarities, mechanisms of regulation and partially overlapping functions [24]. Further, synthetic lethal relationships exist between ATM and DNA-PKcs [26, 27]. Given the intertwined relationships between ATM and DNA-PKcs, the hyperfractionated schedule shown to be of added value in the presence of ATM inhibitors [13], was applied here in the presence of DXC, a putative DNA-PK inhibitor. The subcutaneous route of administration was preferred to the oral route (e.g. through drinking water or by a blunt cannula) for its higher dosage precision and lower level of administration-related complications [28, 29].

Unlike previous in vitro studies reporting a radiosensitizing capacity of DXC towards breast tumor initiating cells, no beneficial effect was observed on survival of mice bearing orthotopic GIC-driven GB [7]. On the contrary, important skin lesions were observed after 4 weeks of DXC treatment that led us to discontinue the drug administration. It is unlikely that the observed skin damages were caused by components such as sodium lauryl sulfate present in the DXC formulation used (Bassado), since similar damages were observed when a pure formulation of DXC was administered (Fig. 1f). DXC only treatment did not impact on animal survival.

Preliminary results show that in the absence of DXC, hyperfractionation of radiation (total 7.5 Gy splitted in 30 x 0.25 Gy fractions) conferred significantly improved animal survival as compared to standard 2.5 Gy x 3 fractions. The mechanistic basis for this effect could reside in the inability of very low doses (such as 0.25 Gy) to trigger the DDR resistance mechanism, while creating sufficiently toxic levels of DNA damage to the tumor cells [22, 30]. This effect is under deeper investigation in our laboratory.

In conclusion, our results do not allow extending to the animal setting the radiosensitizing capacity of DXC previously observed towards in vitro cultured tumor initiating cells [7]. Chronic DXC administration, while not showing significant radiosensitizing capacity towards orthotopic GIC-driven gliomas, caused important skin lesions to the animals, thus not supporting the use of this drug in radiosensitization studies.

## Abbreviations

ARRIVE: Animal research reporting of in vivo experiments
DXC: Doxycycline
GB: Glioblastoma
GIC: Glioma initiating cells
H/E: Hematoxylin/eosin
IR: Ionizing radiation
NOD-SCID: Non-obese diabetic-severe combined immunodeficient
s.c.: subcutaneous
